# Supplementary material for: Inflammation rapidly recruits mammalian GMP and MDP from bone marrow into regional lymphatics
Source: eLife. 2021 Apr 8;10:e66190. doi: 10.7554/eLife.66190 (PMC8137144; doi:10.7554/eLife.66190)
Supplement: Supplementary file 1. [file elife-66190-supp1.docx]

**Supplementary File 1**

Histological categorization of human LN biopsies and their SP (%) frequency.

| **Anatomical location of LN** | **Diagnosis** | **Histological subtype** | **SP (%)*** |
| --- | --- | --- | --- |
| Cervical | reactive | Follicular | 0.23 |
| Inguinal | reactive | Follicular | 39.12 |
| Axillary | reactive | Granulomas | 2.15 |
| Axillary | NHL | Low grade | 0 |
| Cervical | NHL | T-NHL | 0 |
| Cervical | reactive | Follicular | 10.41 |
| Cervical | HL | Mixed | 0 |
| Cervical | reactive | Follicular | 5.52 |
| Cervical | reactive | Viral | 0.14 |
| Inguinal | reactive | Granulomas | 1.22 |
| Cervical | NHL | High grade | 0 |
| Cervical | HL | Mixed | 0 |
| Axillary | NHL | High grade | 0 |
| Inguinal | NHL | High grade | 0 |
| Axillary | NHL | Low grade | 0 |
| Cervical | HL | Nodular | 0.1 |
| Cervical | NHL | High grade | 0 |
| Cervical | reactive | Follicular | 0 |
| Cervical | reactive | Follicular | 0.02 |
| Cervical | NHL | T-NHL | 0.09 |
| Cervical | reactive | Viral | 0.02 |
| Mesenterium | HL | HL | 0 |
| Cervical | reactive | viral | 0 |
| Axillary | reactive | Granulomas | 0.03 |
| Axillary | reactive | Viral | 0.02 |
| Cervical | reactive | Follicular | 0 |
| Mesenterium | NHL | Low grade | 0 |
| Cervical | reactive | Follicular | 0.13 |
| Cervical | HL | Mixed | 0.04 |
| Cervical | reactive | Follicular | 0.12 |
| Cervical | reactive | Follicular | 0 |
| Cervical | HL | Mixed | 0 |
| Cervical | reactive | Follicular | 0 |
| Inguinal | HL | Nodular | 0.02 |
| Mesenterium | reactive | Granulomas | 0.72 |
| Cervical | reactive | Follicular | 0.1 |
| Cervical | reactive | Viral | 0 |

| **Anatomical location of LN** | **Diagnosis** | **Histological subtype** | **SP (%)*** |
| --- | --- | --- | --- |
| Axillary | reactive | Follicular | 0 |
| Cervical | NHL | Low grade | 0 |
| Cervical | HL | Nodular | 0 |
| Axillary | HL | Lymphocyte predominium | 0.09 |
| Axillary | reactive | Granulomas | 0.21 |
| Mesenterium | HL | Lymphocyte predominium | 0 |
| Mesenterium | NHL | High grade | 0 |
| Mesenterium | NHL | Low grade | 0 |
| Inguinal | reactive | Follicular | 0.21 |
| Cervical | NHL | T-NHL | 0.92 |
| Cervical | reactive | Follicular | 0.02 |
| Cervical | reactive | Follicular | 0.07 |
| Mesenterium | NHL | High grade | 0 |
| Axillary | reactive | Follicular | 2.38 |
| Mesenterium | reactive | Follicular | 0.16 |
| Cervical | HL | Mixed | 0.02 |
| Cervical | reactive | Follicular | 0.26 |
| Cervical | reactive | Follicular | 0.3 |
| Axillary | reactive | Follicular | 0.03 |
| Inguinal | NHL | T-NHL | 0 |
| Axillary | NHL | High grade | 0 |
| Cervical | NHL | Low grade | 0 |
| Cervical | NHL | Low grade | 0.47 |
| Cervical | HL | Lymphocyte predominium | 7.69 |
| Cervical | reactive | Granulomas | 0.21 |
| Cervical | reactive | Granulomas | 0.04 |
| Cervical | NHL | Low grade | 2.03 |

* 0 represents no significant content of SP cells (<0.01%; limit of sensitivity).
